# Virtual reality roleplays for patients with depression: A user experience evaluation

**DOI:** 10.1016/j.invent.2024.100713

**Published:** 2024-01-19

**Authors:** Steffen Holsteg, Johanna M. Askeridis, Jarek Krajewski, Philip Mildner, Sebastian Freitag, Tobias Müller, Sebastian Schnieder, Annika Gieselmann, André Karger

**Affiliations:** a Clinical Institute of Psychosomatic Medicine and Psychotherapy, Medical Faculty and University Hospital Düsseldorf, Heinrich Heine University Düsseldorf, Moorenstrasse 5, D-40225 Düsseldorf, Germany; b Institute of Experimental Psychophysiology GmbH, Gustav-Poensgen-Strasse 29, D-40215 Düsseldorf, Germany; c Nuromedia GmbH, Schaafenstrasse 25, D-50676 Cologne, Germany; d Department of Clinical Psychology, Heinrich Heine University Düsseldorf, Universitätsstrasse 1, D-40225 Düsseldorf, Germany

**Keywords:** Virtual reality, Depression, Psychodynamic psychotherapy, User experience, Roleplay, Interpersonal conflict

## Abstract

**Background:**

Virtual reality (VR) has been used successfully and effectively in psychotherapy for a variety of disorders. In the field of depression, there are only a few VR interventions and approaches. Although simple social interactions have been successfully modeled in VR for several mental disorders, there has been no transfer to the field of depression therapy. VR may be employed for psychodynamic psychotherapy to work on interpersonal conflict patterns. In this study, we developed and evaluated a VR intervention for the simulation of roleplay situations in the context of supportive-expressive therapy.

**Methods:**

We conducted a clinical user experience (UX) study at a psychotherapeutic clinic in Düsseldorf, Germany. Eight inpatients with depression and four therapists were included. Semi-structured interviews and qualitative content analysis were used to identify UX issues of the developed VR intervention. Usability questionnaires and technical usage data were also considered. The VR intervention consisted of two therapist-controlled roleplay scenarios designed to support work on the core conflictual relationship theme by allowing patients to interact in typical problematic social situations. Recorded VR roleplays allow for therapeutic debriefing with a change of perspective. Therapists were given the option of using the roleplay in multiple sessions.

**Results:**

All therapists conducted one session per patient with the VR intervention. From the patient interviews, 26 UX issues were extracted, of which one technical malfunction and two unclarities in the interaction with the VR agent were rated as major problems. From the therapist interviews, 14 UX issues were extracted, of which five were rated as major problems related to the interface in the dialog control or the complex system setup.

**Conclusion:**

The main problem was designing a dialog structure that allows both complex conversational flows and a clear control interface. In principle, VR roleplays could be integrated well and safely into therapy. The VR intervention shows promise for providing an emotional experience of interpersonal conflict patterns in the context of psychotherapy. Additionally, other roleplay situations involving various social problem areas must be created and evaluated in terms of the fit to the patients' core conflictual relationship themes.

## Highlights

• Achieving emotion-driven social interactions in VR with depression patients.
• VR roleplays could be integrated well and safely into psychodynamic therapy.
• Developing a complex and easy-to-use dialog structure is a challenge.

## 1 Introduction

Digitization and the use of new technologies in the fields of medicine and psychology are in continuous progress. E-health services have become an indispensable part of modern treatment settings and open new perspectives for innovative and progressive interventions - also in psychotherapy. Virtual Reality (VR) is an innovative technology in the e-health spectrum and is defined as a computer-generated, three-dimensional, interactive environment that simulates reality as closely as possible by mimicking physical laws and sensory impressions (Doerner et al., 2022). One process relevant to the effectiveness of VR applications is that immersive VR environments give users a sense of presence, the illusion of being located inside the rendered virtual environment (Slater, 2009). Through its use in the entertainment and gaming industries, VR is benefiting from constant technical development, a growing range of products, better graphics, new technical features, and decreasing acquisition costs (Dörner et al., 2016). VR therefore offers good conditions and interesting opportunities for use in healthcare interventions.

VR has been studied in the field of psychotherapy since the 1990s (e.g., North et al., 1996) and has been shown to be an effective and successful tool for a variety of disorders (Freeman et al., 2017; Wiebe et al., 2022). In particular, most evidence exists for the use of VR exposure therapy in anxiety disorders, posttraumatic stress disorder and addiction disorders (Emmelkamp and Meyerbröker, 2021; Wiebe et al., 2022), but diverse and new interventions and promising approaches are being developed. In their recent review, Wiebe et al. (2022) also present therapy studies in eating disorders, schizophrenia, attention deficit disorder, and obsessive-compulsive disorder, and highlight the potential for cognitive training in dementia and social skills training in autism spectrum disorder. In the area of depression, Wiebe et al. (2022) identified only 16 therapy studies, which is consistent with the general finding that there has been little research on VR interventions in the area of depression (Lindner et al., 2019).

Taking a closer look at existing VR interventions used in the context of depression therapy, three categories of VR interventions can be identified. First, a simple way to use VR in depression therapy is to offer VR games from other application areas or commercial off-the-shelf products from the entertainment sector to depression patients. For example, VR motor, cognitive or balance training has a positive effect on depression symptoms (House et al., 2016; Yang et al., 2017). These VR games mainly create a motivating, activating, and exciting experience. The use of VR entertainment is also an effective method to distract from unpleasant treatment situations and physical pain, especially in the medical field and psycho-oncology (Sansoni et al., 2022).

Second, VR can be used to immerse patients in a positive and engaging environment, replacing reality with a VR environment through the principle of substitution. Interventions in this category can be further differentiated according to the degree of interaction with the VR environment. Non-interactive VR interventions use the VR environment to perform, for example, mindfulness or relaxation exercises (Flores et al., 2018; Shah et al., 2015). VR facilitates imagination and blocks out environmental stimuli. Most existing VR interventions for depression place patients in interactive, positive environments where simple tasks must be completed. VR interventions address virtual gardening (Rutkowski et al., 2021; Szczepańska-Gieracha et al., 2021), behavioral activation (Colombo et al., 2022; Paul et al., 2022), or supporting exploratory behavior by using a standardized environment (Chen et al., 2020; Habak et al., 2020; Li et al., 2021) or an individual positive place from the patients' autobiographical memories (Fernandez-Alvarez et al., 2021).

Third, some approaches use the technical possibilities of VR to create new VR-unique experiential spaces and innovative new treatment methods. Falconer et al. (2016) showed a reduction in depression severity and self-criticism and an increase in self-compassion using VR-based compassion training for depression patients. Employing an embodiment paradigm, patients first comfort a crying virtual child (show compassion), and then become the child's VR avatar and experience their own compassion in the form of a perspective shift. This technical possibility of virtual perspective change and embodiment into another VR avatar is a promising approach that can be used in other contexts as well (Osimo et al., 2015).

What these interventions have in common is that they are mostly based on cognitive-behavioral approaches. Psychodynamic psychotherapy, with its special focus on working on interpersonal conflicts and relationship patterns (Blagys and Hilsenroth, 2000; Steinert et al., 2017), may also provide a perspective for the use of VR interventions. An example of a manualized psychodynamic therapy approach that focuses on working with interpersonal problems is Luborsky's (1984) supportive-expressive therapy (SET). One of the central concepts of this approach is to identify and address the Core Conflictual Relationship Theme (CCRT; Luborsky et al., 1994). Within the CCRT, symptoms, conflicts, and transference are brought together by prototypically identifying a wish, a response from the other and a response from the self when looking at interactions involving others. Although it cannot be assumed that there is a single characteristic CCRT for patients with depression, typical CCRTs can be identified (Vanheule et al., 2006). For example, the patient's wish to feel happy and to be loved by a significant other is in conflict with the fearful expectation that the other will dislike and ignore the patient. In response, the patient withdraws from the other and, in the sense of a self-fulfilling prophecy, the other comes to dislike the patient (Mark et al., 2003; Vanheule et al., 2006).

VR could be a promising tool to enhance psychotherapy sessions with a realistic and emotionally triggering experience of one's own maladaptive relationship patterns in contact with VR agents, and to enable the exploration of new behavioral patterns. Although many studies have successfully presented simple social interactions and situations in VR, such as exposure therapy for social anxiety disorder (Carl et al., 2019; Wiebe et al., 2022) or social skills training for autism spectrum disorder (Mesa-Gresa et al., 2018), there has been no meaningful transfer of VR social interaction training to patients with depression.

In the current study, we developed a VR-based social interaction training for depression therapy, which was evaluated in a clinical study in terms of usability and user experience (UX) by patients and therapists. Based on the therapeutic orientation of the clinic, the developed intervention was applied in the context of SET. In principle, the intervention represents an element that can be used in conjunction with various therapeutic approaches that work with the reflection of unconsciously repeated relational experiences, such as interpersonal psychotherapy or cognitive behavioral analysis system of psychotherapy.

## 2 Methods

### 2.1 Study design

The present study is a mixed-methods clinical UX study. In line with recommendations for the development of digital health interventions (Kernebeck et al., 2022; Kowatsch et al., 2019), future users were involved in this study at an early stage of development. Both qualitative and quantitative methods were used to have patients and therapists evaluate a newly developed VR intervention. Qualitative UX interviews were conducted with the patients and their therapists. Efficacy-related variables (pre/post measurement) and usability questionnaires were quantitatively collected from patients. As part of the study, a VR mindfulness exercise and a VR roleplay were developed and tested with patients and therapists. The focus of this paper is on the roleplay. The study was approved by the Ethics Committee of the Medical Faculty of Heinrich Heine University Düsseldorf (study no. 2021–1302) and registered in the German Clinical Trials Register (no. DRKS00024981).

### 2.2 Intervention

Due to the therapeutic orientation of the clinic, SET (Luborsky, 1984) was chosen as the basis for the development of the VR intervention, with the work on the CCRT identified as a promising element of SET for transfer to VR. For this purpose, we developed a VR roleplay with two social situations based on typical CCRTs of patients with depression (Beutel et al., 2015; Luborsky, 1984; Vanheule et al., 2006). In creating the roleplay situations, care was taken to keep the starting situations general so that different relationship issues could be represented and the roleplay could be used in other therapy approaches. In these roleplays, we established a semi-autonomous Wizard of Oz system (Pan and Hamilton, 2018) for dialog control, meaning that the therapist controls the course of the interaction by manually selecting the VR agent's behavior and line of dialog from a computer while the patient is in VR. The therapist has two control options via the interface, a dialog tree or buttons containing recommended responses. The dialog lines were reviewed and recorded by professional actors. In addition, video recordings of the dialogs were used to transfer the actors' body language to the VR agents.

**Fig. 1:**
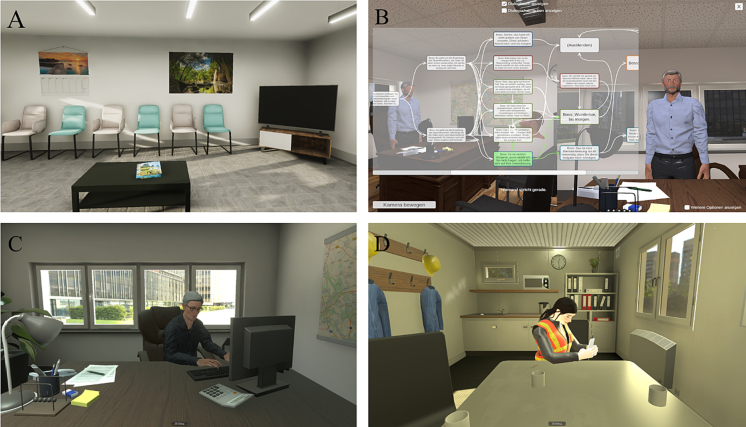
Screenshots of the virtual reality intervention. *Note*: A = Onboarding waiting room environment, B = Therapist dialog control interface, C = Boss roleplay from the patient's perspective, D = Colleague roleplay from the patient's perspective.

Two different processes were used to develop the dialogs: In roleplay 1 (boss situation), there was a complex selection of different reaction modes and many dialog lines whereas in roleplay 2 (colleague situation), there was a stricter course of conversation and therefore fewer reaction modes and dialog lines. These two types of dialog numbers were chosen to capture therapist preferences and usability as part of the study. Furthermore, therapists utilize multiple levels of difficulty to adapt the emotional burden, as well as customizable scenario parameters, such as a choice of different VR agents, to tailor the experience to the patient's CCRT. Both roleplays involve workplace situations, as these are primarily clearly defined areas with unspecific others who, unlike close relatives, are not often associated with concrete relationship experiences. Patients do not need to have worked in the presented workplace in order to perform the roleplays. A more detailed overview of the content of the roleplays is given in Table 1. Screenshots of the VR environment are shown in Fig. 1.

**Table 1:** Content of the Virtual Reality (VR) roleplay.

	Boss situation	Colleague situation
Aim	• Communicating one's own needs • Distancing oneself from the boss's needs • Making a compromise • Dealing with guilt and/or anger	• Active asking for support • Disclosure of personal problems • Dealing with emotional problems caused by anger and rejection
Context	• The patient gets another task from the boss just before he/she was supposed to go home for a private meeting with friends. • Task content: preparing the quarterly figures • Task duration: either 30 min or 2 h, depending on the therapist's choice	• The patient repeatedly arrives late to work and misses a team meeting to assign shifts for the next day. Patient encounters angry coworker. • The colleague should be asked about the assigned shift. However, the patient is unable to attend this shift due to an exceptional personal situation.
VR environment	Waiting room • Waiting room with several chairs, a large window, and a TV screen • Patient fills in short questionnaires • Short introduction to the roleplay situation with imagination	
	Boss's office The boss is working at a large desk in the middle of the room, and the patient is sitting in front of the desk.	Social room of a craft business The colleague is sitting on a table across from the patient, typing on their phone.
VR agent	Four options: male or female, young or old They are dressed in formal attire.	Two options: young male or young female They are wearing a high-visibility safety vest and a safety helmet.
Mode of reaction	The therapist can choose between six modes of reaction, depending on whether the participant accepts or denies to do the task. It is possible to switch between modes at any time. • Acceptance o Friendly appreciation o Lack of appreciation • Denial o Unfriendly denial o Friendly manipulation o Friendly acceptance o Conflict	The therapist can choose between two modes of reaction, angry and empathetic, which can be switched throughout the conversation, depending on whether the participant is reserved or open about their personal problems. • Reserved o Angry: Unfriendly accusations • Open (self-disclosure) o Empathetic: Offering support o Empathetic: Negotiation for support

#### 2.2.1 Intervention components and procedure

The present intervention consists of different contents for the patients, all in a seated VR setup, and a control interface for the therapist on the computer. Patients began with an onboarding phase where they entered a virtual waiting room to familiarize themselves with the environment while the therapist configured the roleplay. Before starting the roleplay, patients completed two short questionnaires in VR and then received an auditory introduction to the roleplay situation, including an imagination of their own similar experience.

In Phase 2, the roleplay was performed. The VR environment changed from the waiting room to the appropriate roleplay's environment and the patients were immersed in a virtual social interaction. The responses of the VR agent were selected by the therapist and the patients spoke normally.

In Phase 3, patients removed the VR headset and a therapeutic debriefing was conducted. The debriefing tool provided the opportunity to watch a recording of the VR roleplay situation together with the therapist. The patient's voice and body movements were captured through the VR headset and provided as a video that could be played through the VR or the therapist's computer. A perspective change function made it possible to adopt any perspective in the video. Patients were represented by a standardized VR avatar (male or female). This video was intended to promote insight into one's own CCRT by looking at the dialog from a different perspective, and to support a conversation about possible and new patterns of behavior.

In Phase 4, the roleplay could then be performed again, e.g., in order to try out new behaviors or to observe one's own (dysfunctional) behavior or relationship patterns more closely. In addition, the system provided the therapists with a reporting function where the results of the VR questionnaires and documentation of the dialog process were available.

Therapists were asked to decide how many therapy sessions they wanted to organize with the roleplays in order to investigate their integration into the therapy sessions and the time needed to carry out the intervention.

### 2.3 VR technology

**Fig. 2:**
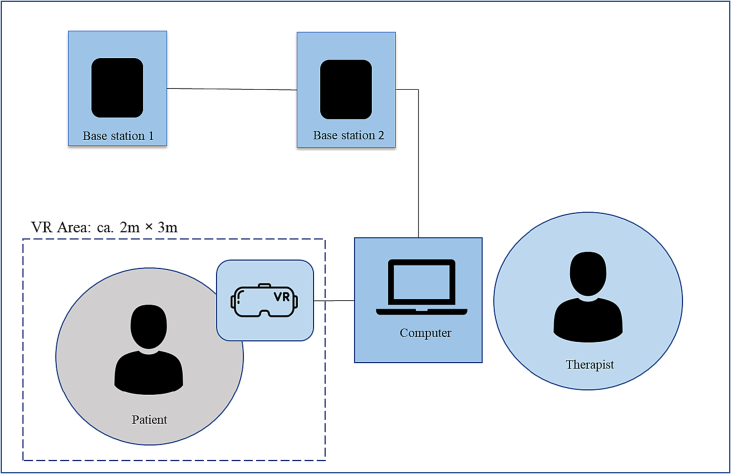
System setup. *Note*: VR = Virtual Reality.

For the VR roleplay, the HTC Vive Pro Eye (HTC Corporation, Taoyuan, Taiwan) was used, which is a tethered VR system where the VR headset is connected to a PC via a cable, and two base stations had to be placed in the room to provide external room-scale tracking of VR headset and controllers. The system setup is shown in Fig. 2. The HTC Vive Pro Eye headset contains two AMOLED screens, with a resolution of 2 × 1440 × 1600 pixels per eye, a refresh rate of 90 Hz, and a field of view of 110°. The headset also includes integrated headphones with surround sound and localization. Hand controllers are used for interacting with the system. Unity3D was used to create the VR intervention.

### 2.4 Eligibility criteria

Patients undergoing inpatient psychotherapy at the Clinic for Psychosomatic Medicine and Psychotherapy of the LVR Clinic Düsseldorf, Germany with a clinical diagnosis of unipolar depression were eligible to participate in the study. The diagnosis of depression was defined according to ICD-10 (World Health Organization, 1992), categories F32 - F34, and was assigned by a psychotherapist as part of the diagnostic process prior to the inpatient stay. The absence of specific suicidal ideations was a requirement for inpatient treatment at the clinic.

Patients could be included in the study if they did not meet any of the following exclusion criteria: (a) limited German language skills, (b) uncorrected visual impairment or visual disturbance, (c) inadequately corrected hearing, (d) known epilepsy, (e) pre-existing neurological or psychological conditions affecting the vestibular organ, affecting the sense of balance, or altering visual perception, (g) current COVID-19 disease, and (f) a body mass index of less than or equal to 17. Criterion f was chosen because the design and body image of the patients' VR avatars were not taken into account during the development of the pilot application and a possible influence on the intervention was suspected. Additionally, to participate in the roleplay, the treating therapist had to approve the content of the VR intervention as appropriate for the patient's CCRT.

Therapists who work at the LVR Clinic Düsseldorf and whose patients participate in the study could be included if they: (a) are physicians or psychologists, (b) have a psychotherapeutic training or are in training, and (c) are psychodynamically oriented.

### 2.5 Procedure and sample selection

**Fig. 3:**
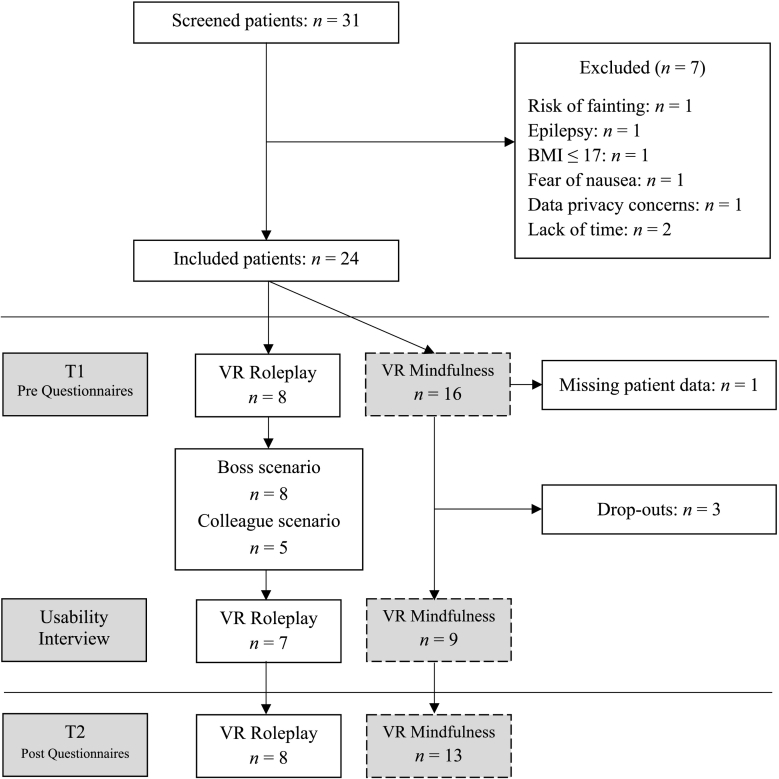
Patient flow chart. *Note*: BMI = Body Mass Index, VR = Virtual Reality.

The study was conducted from June 1, 2021 to November 19, 2021 at the Clinic for Psychosomatic Medicine and Psychotherapy of the LVR Clinic Düsseldorf, Germany. Thirty-one patients were informed about the study with leaflets and a five-minute oral presentation by the study staff. Interested patients were asked to complete a short screening questionnaire and, if suitable, an appointment was made for a personal information interview. Of the thirty-one patients screened, three did not meet the eligibility criteria and four had individual reasons for not participating. The participant flow chart is shown in Fig. 3. After verifying the eligibility criteria, patients were informed of the procedure, content, and objectives of the study and provided written informed consent. Pre-measurement questionnaires and a technical briefing on the VR mindfulness exercise followed for all patients. Patients' therapists were informed of their participation in the study and were able to schedule the roleplay sessions around their therapy.

A total of twenty-four patients were enrolled in the study. Of these, eight underwent the VR roleplay. This was mainly due to the suitability of the patient's CCRT for the roleplay and the willingness of the treating therapist to incorporate the VR intervention into the therapy. A total of five therapists at the clinic were eligible to use the roleplays, with one therapist declining to participate in the study.

The VR technology for the roleplay session was set up in advance by study staff in a separate room. The VR roleplay was controlled and directed independently by the therapists after 30 min of individual training in the use of the program. The roleplay consisted of two scenarios; the boss scenario was used for all eight patients and the colleague scenario for five patients.

After using the VR intervention, patients completed a brief usability questionnaire, and a qualitative semi-structured UX interview about the roleplay was conducted with patients and therapists by telephone. For the interview, seven patients were available. There were no dropouts among patients and therapists who used the roleplay.

In the study, sixteen patients also used a VR mindfulness module as an adjunctive self-management intervention. Four of these patients used both applications, the roleplay and the mindfulness exercises. A separate qualitative interview was conducted with patients who used the VR mindfulness exercise. At the end of the inpatient stay, a final appointment was scheduled for patients to complete the post questionnaires. The focus of this paper is on the VR roleplay; therefore, data from the eight patients who used the roleplay constitute the final sample.

### 2.6 Data collection

This paper includes data from three different sources: Qualitative data from UX interviews, quantitative questionnaires completed at the beginning and end of the study as well as after completion of the VR intervention, and technical usage data. The VR system recorded the duration of roleplays, configurations, and dialog history (selected dialog lines).

#### 2.6.1 User experience interview

Semi-structured UX interviews were conducted with patients and therapists immediately after using the system. Due to pandemic restrictions, these were conducted by telephone and included printed images of the VR environment and system setup. The patient interviews consisted of 30 questions covering the following categories: well-being, previous technical experience, headset comfort, VR questionnaire handling, onboarding, roleplay experience, debriefing, and overall system evaluation. The therapist interviews consisted of 39 questions in the following categories: previous technical experience, side effects, system setup, use of the system, and overall system evaluation.

#### 2.6.2 Measures

The self-developed screening questionnaire asked about the study's eligibility criteria and reasons for non-participation. Over the course of the study, patients completed various self-report measures. An overview of the questionnaires used in the overall study is provided in Appendix A. The questionnaires relevant to this publication are outlined below.

Pre-measurement included a demographic questionnaire that also assessed diagnosis and previous experience with VR. Patients also completed the Beck Depression Inventory II (BDI-II; Beck et al., 1996) and the Beck Anxiety Inventory (BAI; Beck et al., 1988) as pre-post measures. The BDI measures the severity of depressive symptoms using 21 items rated on a four-point scale (“not at all” to “severely”). The BAI measures the expression of anxiety-related symptoms, also with 21 items and a four-point scale. The German versions of the BDI-II and the BAI have high reliability and construct validity (Geissner and Huetteroth, 2018; Kühner et al., 2007). BAI and BDI-II severity scores are based on the total score.

The Post System Usability Questionnaire (PSSUQ; Lewis, 1995) and the Satisfaction with Inpatient Care Questionnaire (ZUF-8; Schmidt et al., 1989) were also administered as part of the post-measurement. The PSSUQ measures the user satisfaction with a technical system with a high reliability and sensitivity (Lewis, 2002). It was translated into German and contains 19 statements about the usefulness of the system, the information quality, and the interface quality, allowing for a separate evaluation of these subscales (Sauro and Lewis, 2016). The rating is based on a seven-point scale (“strongly disagree” to “strongly agree”). The ZUF-8 assesses overall treatment at the clinic, using eight items rated on a four-point scale (“very dissatisfied” to “very satisfied”).

Furthermore, patients completed the After Scenario Questionnaire (ASQ; Lewis, 1991) after the VR intervention. The ASQ is a short form of the PSSUQ that measures technical usability immediately after a scenario, consisting of three items, also rated on a seven-point scale. The ASQ was translated into German and supplemented with a question about overall satisfaction with the type of exercise conducted (“Please rate how much you liked the scenario overall”).

### 2.7 Data analysis

#### 2.7.1 Quantitative analysis

Due to the small sample size of eight patients, the questionnaire scores and technical usage data were analyzed descriptively.

#### 2.7.2 Qualitative analysis

To categorize and analyze any UX issues, a content-structuring qualitative content analysis following (Mayring, 2022) was conducted. A category system was developed using a deductive, literature-based approach. The three main groups of VR usability problems called VR environment, device interaction, and task-specific identified by Geszten et al. (2018) formed the main frame of the category system.

Thirty-three VR usability problems and VR evaluation heuristics were extracted from previous literature. This yielded twelve problems from Geszten et al. (2018), twelve heuristics from Sutcliffe and Gault (2004), and nine heuristics from Murtza et al. (2017). Similar problems and heuristics were merged, resulting in 21 of the candidates being merged into seven categories. Of the remaining twelve candidates, five were kept as they were, three were modified, and four were deleted due to inapplicability to the intervention. One task-specific category was added. All definitions were reformulated for the sake of consistency. The pilot category system consisted of 16 categories, nine VR environment categories, six device interaction categories, and one task-specific category. Coding rules as well as examples were added to the categories.

Two interviews were coded independently by two coders who then discussed any difficulties and disagreements, resulting in a reworked codebook. The process was repeated, this time coding all interviews collected for the study, meaning interviews on both the roleplays and the mindfulness exercises as well as with patients and therapists. One device interaction category and one task-specific category were added.

To analyze the results, the coded statements were extracted per category. Similar statements were subsumed under one issue. Each issue was rated for both severity as well as potential impact on patient safety, in accordance with Peute et al. (2013). Severity was rated using Nielsen's (1994) scale (0 = no problem, 1 = cosmetic problem, 2 = minor problem, 3 = major problem, 4 = catastrophe). The item “no problem” was not included as it technically does not judge a problem's severity (Herr et al., 2016). Potential impact on patient safety was rated binarily (0 = no impact, 1 = potential impact). Two raters discussed and agreed on each rating together.

## 3 Results

### 3.1 Sample characteristics

The eight patients in the final sample had a mean age of 35.88 years (*SD* = 13.85, range 19–58). Four of them were male, three female and one diverse. Seven reported depression as their primary diagnosis and one patient reported an anxiety disorder. Three patients had previous VR experience. In addition, four male therapists aged 29 to 37 (*M* = 33, *SD* = 3.16) were interviewed as part of the study. All of them were physicians in psychotherapeutic training.

### 3.2 Technical usage data

For all eight patients, therapists designed a therapy session (50 min) using the roleplay. All roleplays were completed, there were no early exits from roleplay situations. The average duration of the boss scenario was *M* = 5:30 min (*SD* = 0:42). All eight patients performed the boss scenario, and two patients performed it a second time. The colleague scenario lasted a mean of *M* = 6:59 min (*SD* = 0:27) and was performed by five patients; there were no repetitions. All therapists used the dialog tree to control the VR roleplay.

During the dialog process, the friendly manipulative reaction mode was used eight times in combination with the conflict mode in the ten boss roleplays. In one roleplay, only the friendly appreciation mode was used. In addition, one dialog course consisted mainly of lack of appreciation and conflict dialog lines. In four of the five colleague roleplays, the dialog process began with several unfriendly accusations from the colleague; in one roleplay, the colleague was immediately in empathetic response mode. In all five colleague roleplays, there was a negotiation for helpful support.

### 3.3 Quantitative evaluation

Regarding efficacy-related variables for depression and anxiety, the eight patients who used the VR roleplay showed a descriptive decrease from pre- to post-measurement in BDI-II score (Pre: *M* = 21.38, *SD* = 8.23; Post: *M* = 14.50; *SD* = 10.18) and BAI score (Pre: *M* = 15.38, *SD* = 9.96; Post: *M* = 12.38, *SD* = 8.91).

The mean PSSUQ score was *M* = 4.82 (*SD* = 0.68, *n* = 7) for system usefulness and *M* = 4.33 (*SD* = 0.93, *n* = 8) for interface quality. Only three patients completed the information quality scale, and the mean score was *M* = 4.05 (*SD* = 0.73). The mean score of the ZUF-8 was *M* = 27.57 (*SD* = 2.76, *n* = 7).

The evaluation of the VR roleplays immediately after their implementation showed an ASQ mean of *M* = 4.42 (*SD* = 1.04, *n* = 8) for the boss scenario, with an overall rating of *M* = 4.25 (*SD* = 0.89). The colleague scenario received an ASQ mean of 4.20 (*SD* = 1.28, *n* = 5), with an overall rating of *M* = 4.20 (*SD* = 0.84).

### 3.4 Qualitative content analysis

The focus of the qualitative content analysis was on identifying UX problems, but positive aspects were also mentioned in the interviews that were not classified. For example, all seven patients described a positive overall impression and six perceived the VR roleplays as a helpful addition to therapy; one patient expressed concerns about whether he would have performed the same in real life situations.

The final category system consisted of 18 categories, nine VR environment categories, seven device interaction categories, and two task-specific categories (Appendix B). All interviews were re-coded using the final codebook. To provide an account on inter-rater reliability, α of Krippendorff (1970) was computed using the SPSS macro by Hayes and Krippendorff (2007). Αn α ≥ 0.800 is often required (Krippendorff, 2004), which was achieved on the first round of coding with the final codebook for the therapist interviews (α = 0.886, 95 % CI [0.715, 1.00]) and on the second round for the patient interviews (α = 0.850, 95 % CI: [0.756, 0.944]). Any remaining disagreements were discussed and resolved.

#### 3.4.1 Patients

A total of 26 UX issues were identified from the qualitative interviews with the seven patients. The individual UX issues, along with their severity and potential impact on patient safety ratings, as well as a mapping of the UX issues to the respective patients, are listed in Table 2. The UX issues are distributed among the different categories as follows: 15 issues were assigned to the VR environment group, seven to the device interaction group, and seven to the task specific group, with three issues assigned to two groups.

**Table 2:** User experience issues of patients, including their categories, severity and safety ratings, and distributions among patients.

Group	Category	Issue	Severity rating	Safety rating	*n* of patients affected	Pa	Pb	Pc	Pd	Pe	Pf	Pg
VR environment	E1: Natural engagement	Lack of visible hands and feet on patient avatar	1	0	1						X	
	E1a: Natural environment and perception	Situational aspects of the boss situation did not completely correspond to the patient's expectations	1	0	1			X				
		VR waiting room was perceived to look blurry	1	0	1					X		
		Volume of agent's voice lines was not optimal	2	0	2					X	X	
		Unrealistic environmental details	1	0	2						X	X
	E1b: Natural interaction	Agent movements were perceived to be artificial	1	0	1	X						
		Affirmative interjections were perceived as interruptions	1	0	1					X		
	E2: Presence	Therapist's audible actions in the same room interfered with presence	1	0	2		X			X		
		Difficult to put oneself in the situation and act authentically	2	0	2	X				X		
		Environment was perceived to be artificial	1	0	1							X
	E3: Co-presence	Colleague reactions ignored shared knowledge	3	0	1		X					
		Colleague dialog lines were perceived to be artificial	2	0	1							X
	E8: Clear turn-taking	Unclear who would begin the conversation (patient or agent)	2	0	2		X		X			
		Unclear whose turn it was to talk because turn-taking pauses were too long	3	0	3		X				X	X
		Agent's pauses on turn-taking were perceived to be too long to be natural	2	0	5	X	X	X			X	X
Device interaction	D1b: Controls	Controls using controller was disliked	1	0	2			X				X
	D2: Learnability	Proper way of acting in the VR environment was unclear	2	0	3			X			X	X
	D4: Headset comfort	Headset was too heavy	2	0	4		X	X	X	X		
		Eyes had to get used to VR	1	0	1					X		
		Sound was too quiet due to bad headset fit	1	0	1							X
	D6: Glitchiness	Malfunctioning replay of roleplay recording	3	0	5	X	X	X	X			X
		Malfunctioning audio output during introduction	2	0	1			X				
Task-specific	T1: Fit for purpose	Uncomfortable because of the roleplay situation	1	1	5	X	X	X	X		X	
		Difficult to put oneself in the situation and act authentically	2	0	5	X		X		X	X	X
		Uncomfortable because of the voice recording	1	0	2	X					X	
		Colleague reactions ignored shared knowledge	3	0	1		X					
		Affirmative interjections were perceived as interruptions	1	0	1					X		
		Unclear consequences of negotiation outcome in the boss situation	1	0	1			X				
	T2: Fit for context	Therapist's audible actions in the same room interfered with presence	1	0	2		X			X		

*Note*: VR = Virtual Reality, Severity Rating: Nielsen scale (1–4; Nielsen, 1994), Safety Rating: Potential impact on patient safety, binary (0/1), P*x*: Individual patient (X = affected by issue).

Of the 26 UX issues, three were rated as major problems in the severity rating. A total of five patients reported problems with the replay of the roleplay recordings during debriefing. Three patients mentioned that there were too many pauses during the roleplay, which resulted in patients not knowing if they needed to say something. In addition, one patient reported that shared knowledge was ignored by the colleague during the roleplay. No issues were rated as catastrophic. The UX issue of patients feeling uncomfortable due to the roleplay situation was mentioned by five patients and was rated as potentially impacting patient safety. All other patient UX issues were rated as not impacting patient safety.

#### 3.4.2 Therapists

A total of 14 UX issues were identified in the qualitative interviews with the four therapists. These, along with ratings of severity and potential impact on patient safety, as well as the distribution of UX problems among the therapists who mentioned them, are presented in Table 3. Of the 14 UX issues, one was assigned to the VR environment group, ten to the device interaction group, and three to the task specific group. All issues were allocated to one group.

**Table 3:** User experience issues of therapists, including their categories, severity and safety ratings, and distributions among therapists.

Group	Category	Issue	Severity rating	Safety rating	*n* of therapists affected	Ta	Tb	Tc	Td
VR environment	E2: Presence	Difficult for patient to put themselves in the situation	2	0	1		X		
Device interaction	D1: User interface design	Clicking onto the wrong dialog line	3	0	1	X			
	D1a: Interface	Difficult to navigate the dialog tree	3	0	2			X	X
		Unclear information on system status	2	0	2			X	X
	D2: Learnability	Orientation time was necessary	2	0	1	X			
		Unaware of alternate dialog navigation interface	2	0	1		X		
	D4: Headset comfort	Headset was too tight	1	0	1				X
		Blurry field of view	1	0	1				X
	D6: Glitchiness	Malfunction of dialog lines	3	0	2	X		X	
		Dialog speech output not interruptible	2	0	1			X	
	D7: System setup	Setting up the system perceived as a burden	3	0	1				X
Task-specific	T1: Fit for purpose	Difficult for patient to handle the roleplay situation	2	1	1		X		
		Lack of dialog options for colleague	3	0	2		X		X
		Lack of very mean dialog options for boss	2	0	1				X

*Note*: VR = Virtual Reality, Severity Rating: Nielsen scale (1–4; Nielsen, 1994), Safety Rating: Potential impact on patient safety, binary (0/1), P*x*: Individual therapist (X = affected by issue).

Five of the 14 UX issues mentioned were rated as a major problem. In the area of interface design, one therapist cited clicking on wrong dialog lines and two cited difficulties in navigating the dialog tree. In addition, malfunctioning dialog lines (*n* = 2) were an issue in the area of glitchiness. Furthermore, the system setup was perceived as a burden (*n* = 1) and missing dialog lines in the colleague roleplay were criticized (*n* = 2). No issues were classified as a catastrophe. The issue of patients having difficulty handling the roleplay situation (*n* = 1) was rated as potentially influencing patient safety.

## 4 Discussion

The study's aim was to investigate the conception and application of a VR intervention within psychodynamic psychotherapy. A therapist-controlled VR roleplay for working on the CCRT was developed. Semi-structured UX interviews were conducted with patients and therapists to identify UX issues. In addition, the usage data in combination with the UX issues provide important information for the development of the intervention.

In general, the interviews showed that the VR intervention was well-received and that patients found the roleplay to be a valuable addition to therapy. The quantitative evaluation of the PSSUQ showed that the system usefulness subscale was rated higher than the interface quality subscale, suggesting a closer look in the context of the UX interviews. The information quality subscale was completed by only a few patients because no error messages occurred. Based on the ZUF-8, satisfaction with the overall treatment is in the upper range.

The in-context application showed that the design of an entire therapy session with the VR intervention prevailed and that there was no repeated use of the intervention in another therapy session. This may have been caused by the complex system setup or the highly specific roleplay situations. Alternatively, the limited number of dialog lines may result in a limited replay value. As a result, therapists are considered infrequent users of the VR application, which places higher demands on the UX because there is often more time between VR sessions. Usage data also show that therapeutic debriefing of the roleplay situations with the Perspective Shift feature is an important component of the intervention.

Several UX issues were identified in different areas of the category system, with varying degrees of severity. In particular, the design of the VR environment revealed several cosmetic issues that could be fixed with minor revisions. On the other hand, technical problems with the replay of the recorded roleplays or malfunctioning dialog lines were major issues that need to be fixed.

Looking at the other minor and major UX issues experienced by patients and therapists, interrelationships and interactions become apparent. One group of issues is related to the design of human social interactions in VR. Using a semi-autonomous Wizard of Oz system means that content and dialog lines have to be developed in advance. During the development process, this difficulty became apparent and led to the choice of two different approaches to dialog creation. The complex dialog structure of the boss scenario, with many reaction options, resulted in a more confusing dialog tree and was harder to control. The less complex colleague scenario resulted in a slower conversation due to the lack of dialog lines. Therapists were particularly dissatisfied with the number of dialog lines in the colleague scenario, leading to a longer selection phase for the appropriate dialog lines; the resulting pauses were perceived by patients as too long for a natural interaction.

The interviews show that the boss roleplay was preferred. The system revision should therefore focus on improving the interface usability. A better structuring and summary of the dialog lines would be conceivable. Future directions could include the use of artificial intelligence (AI) to support control. A recommender system for dialog lines would be obvious. However, a fully AI-controlled autonomous VR agent should be considered with caution in terms of patient safety. Therapist control is an important component, especially with regard to triggering traumatic events. The use of specific AI systems, e.g., for facial mimicry mirroring or speech recognition and analysis, could make interactions with the VR agent much more realistic (Pan and Hamilton, 2018), even under therapist control.

Another problem area is the system setup, which was described as a burden by the therapists and was therefore undertaken by study staff. The tethered VR system shows its weaknesses here, because it requires significantly more effort in preparation and led to the therapists hesitating to use the VR system at all. Extensive training and familiarization with the system is therefore important. In the future, the technical development of VR systems may make the setup easier, or a switch to a stand-alone system may be considered, which still has disadvantages in the area of graphical realism.

Despite the problems identified, the picture that emerges is generally positive for the use of VR roleplays in psychodynamic psychotherapy. In spite of minor problems affecting presence, it was shown that an immersive, reality-based social situation can be created via the VR system. It is conceivable that the VR intervention leads to more insight into one's own CCRT through the emotional experience of one's own interpersonal conflict in the roleplay situation, similar to Beutel et al. (2019) emotion-focused psychodynamic psychotherapy. Future research should investigate the underlying effect. In addition, the VR intervention expands the repertoire of digital interventions and may serve as a complementary module for digital psychodynamic or interpersonal interventions (e.g., Beutel et al., 2018).

### 4.1 Impact on patient safety

Two issues were identified as potentially influencing patient safety but on closer inspection were considered to be minor. First, a large proportion of patients were agitated and uncomfortable prior to the roleplay. This was partly due to the roleplay situation, as the patients did not know what to expect, and partly due to a lack of experience with VR. Second, one therapist indicated that the roleplay situation was potentially overwhelming. Patients did not express such an experience in the interviews. Both aspects underline the importance of the therapist's presence and conducting of the roleplay. By acting as a known reference person, the patient's reactions can be assessed and the dialogs can be controlled accordingly. The influence of the intervention on the therapeutic alliance is an interesting aspect for future research.

Furthermore, the interviews did not reveal any issues in the category of simulator sickness (E4). In addition, there were no indications of side effects of the intervention in the questionnaires, including the ASQ. It can therefore be assumed that this VR intervention is safe.

### 4.2 Limitations

In the present study, the developed VR intervention was only evaluated in a small group of patients and therapists to provide initial findings for further development. In addition to the small sample size, the inclusion and exclusion criteria did not define comorbid disorders, and patient access to the roleplays depended on therapists' willingness to integrate the VR technique into their therapy sessions, further limiting the sample and data set. Additionally, the course of the conversations in the roleplay situations depended on the therapists' choice of response options and VR agents and therefore may vary between practitioners. Furthermore, the influence of the roleplays on the therapeutic process was not investigated in the present study.

In addition, the VR intervention was a pilot with only two roleplay scenarios, both designed for the context of the work environment. To open the intervention to a broader range of patients, more scenarios from different life domains need to be implemented and different CCRTs need to be addressed. Finally, the roleplays required the patients to put themselves in a specific situation that they may not have experienced in their personal lives. This requires the patients to be able to imagine these situations and to be willing to engage in them.

### 4.3 Recommendations for future research

The results suggest that the developed VR intervention should be investigated in future research. In addition to adapting the intervention based on the study results, the main goal should be to obtain a more meaningful sample for further studies, suitable for investigating efficacy in feasibility and randomized controlled trials. These studies should also evaluate the mechanism of efficacy. Fitting to therapy topics, effects on the therapeutic alliance, recording of roleplay situations for expert rating, and assessing the fit of the available dialog lines would also be useful additions to future research.

In the present study, the VR intervention was used in the context of SET, but it is assumed that the roleplay can also be used to work on interpersonal experiences in other therapeutic approaches. The application in other therapeutic approaches and the fit of the roleplay situations with other theoretical derivations is also a perspective for future research.

## 5 Conclusion

This study presents a new VR intervention designed to enrich the psychotherapeutic work with patients suffering from depression. The integration of VR roleplays into therapy sessions is possible and has been accepted. Several UX problems became apparent in the interviews with therapists and patients. A central problem appears to be bringing together a roleplay's complex dialog structure with an easy-to-use interface. In principle, the use of immersive roleplays to generate emotion-driven social interactions represents an interesting perspective for working on interpersonal conflict patterns in psychodynamic psychotherapy or other therapeutic approaches that focus on relational experiences. Further development of the VR intervention seems appropriate.

## Abbreviations

AI: (Artificial Intelligence)
VR: Virtual Reality
SET: Supportive-Expressive Therapy
CCRT: Core Conflictual Relationship Theme
UX: User Experience

## Funding

This work was supported by EFRE NRW 2014–2020 (Leitmarkt.Agentur NRW) through the project DeepVR - Virtual Reality-based support of acute therapy and relapse prophylaxis in the treatment of unipolar depression (funding ID: EFRE-0801333, LS-2-1-020c).

## Declaration of competing interest

The authors declare that they have no known competing financial interests or personal relationships that could have appeared to influence the work reported in this paper.

## Appendices Supplementary data

Appendix A Questionnaires during the study. Appendix A

Appendix B Category system. Appendix B

## References

[bb0005] Beck A.T., Epstein N., Brown G., Steer R.A. (1988). An inventory for measuring clinical anxiety: psychometric properties. J. Consult. Clin. Psychol..

[bb0010] Beck A.T., Steer R.A., Ball R., Ranieri W. (1996). Comparison of Beck Depression Inventories -IA and -II in psychiatric outpatients. J. Pers. Assess..

[bb0015] Beutel M.E., Barthel Y., Haselbacher A., Leuteritz K., Zwerenz R., Imruck B.H., Brähler E. (2015).

[bb0020] Beutel M.E., Böhme K., Banerjee M., Zwerenz R. (2018). Psychodynamic online treatment following supportive expressive therapy (SET): therapeutic rationale, interventions and treatment process. Z. Psychosom. Med. Psychother..

[bb0025] Beutel M.E., Greenberg L., Lane R.D., Subic-Wrana C. (2019). Treating anxiety disorders by emotion-focused psychodynamic psychotherapy (EFPP)-an integrative, transdiagnostic approach. Clin. Psychol. Psychother..

[bb0030] Blagys M.D., Hilsenroth M.J. (2000). Distinctive features of short-term psychodynamic-interpersonal psychotherapy: a review of the comparative psychotherapy process literature. Clin. Psychol. Sci. Pract..

[bb0035] Carl E., Stein A.T., Levihn-Coon A., Pogue J.R., Rothbaum B., Emmelkamp P., Powers M.B. (2019). Virtual reality exposure therapy for anxiety and related disorders: a meta-analysis of randomized controlled trials. J. Anxiety Disord..

[bb0040] Chen K., Barnes-Horowitz N., Treanor M., Sun M., Young K.S., Craske M.G. (2020). Virtual reality reward training for anhedonia: a pilot study. Front. Psychol..

[bb0045] Colombo D., Suso-Ribera C., Ortigosa-Beltrán I., Fernández-Álvarez J., García-Palacios A., Botella C. (2022). Behavioral activation through virtual reality for depression: a single case experimental design with multiple baselines. J. Clin. Med..

[bb0050] Doerner R., Broll W., Grimm P., Jung B. (2022). Virtual and Augmented Reality (VR/AR): Foundations and Methods of Extended Realities (XR). Springer Nature.

[bb0055] Dörner R., Broll W., Grimm P., Jung B. (2016). Virtual und Augmented Reality (VR/AR)–Auf dem Weg von der Nische zum Massenmarkt. Informatik-Spektrum.

[bb0060] Emmelkamp P.M.G., Meyerbröker K. (2021). Virtual reality therapy in mental health. Annu. Rev. Clin. Psychol..

[bb0065] Falconer C.J., Rovira A., King J.A., Gilbert P., Antley A., Fearon P., Brewin C.R. (2016). Embodying self-compassion within virtual reality and its effects on patients with depression. Br. J. Psychiatry.

[bb0070] Fernandez-Alvarez J., Colombo D., Suso-Ribera C., Chirico A., Serino S., Di Lernia D., Botella C. (2021). Using virtual reality to target positive autobiographical memory in individuals with moderate-to-moderately severe depressive symptoms: a single case experimental design. Internet Interv..

[bb0075] Flores A., Linehan M.M., Todd S.R., Hoffman H.G. (2018). The use of virtual reality to facilitate mindfulness skills training in dialectical behavioral therapy for spinal cord injury: a case study. Front. Psychol..

[bb0080] Freeman D., Reeve S., Robinson A., Ehlers A., Clark D., Spanlang B., Slater M. (2017). Virtual reality in the assessment, understanding, and treatment of mental health disorders. Psychol. Med..

[bb0085] Geissner E., Huetteroth A. (2018). Beck Anxiety Inventory deutsch–Ein reliables, valides und praxisgeeignetes Instrument zur Messung klinischer Angst. Psychother. Psychosom. Med. Psychol..

[bb0090] Geszten D., Komlodi A., Hercegfi K., Hamornik B., Young A., Koles M., Lutters W.G. (2018). A content-analysis approach for exploring usability problems in a collaborative virtual environment. Acta Polytech. Hung..

[bb0095] Habak S., Bennett J., Davies A., Davies M., Christensen H., Boydell K.M. (2020). Edge of the present: a virtual reality tool to cultivate future thinking, positive mood and wellbeing. Int. J. Environ. Res. Public Health.

[bb0100] Hayes A.F., Krippendorff K. (2007). Answering the call for a standard reliability measure for coding data. Commun. Methods Meas..

[bb0105] Herr, S., Baumgartner, N., & Gross, T. (2016). Evaluating severity rating scales for heuristic evaluation. Proceedings of the 2016 CHI Conference Extended Abstracts on Human Factors in Computing Systems.

[bb0110] House G., Burdea G., Grampurohit N., Polistico K., Roll D., Damiani F., Demesmin D. (2016). A feasibility study to determine the benefits of upper extremity virtual rehabilitation therapy for coping with chronic pain post-cancer surgery. Br. J. Pain.

[bb0115] Kernebeck S., Scheibe M., Sinha M., Fischer F., Knapp A., Timpel P., Vollmar H. (2022). Development, evaluation and implementation of digital health interventions (part 1)-discussion paper of the digital health working Group of the German Network for Health Services Research (DNVF). Gesundheitswesen.

[bb0120] Kowatsch T., Otto L., Harperink S., Cotti A., Schlieter H. (2019). A design and evaluation framework for digital health interventions. IT-Information Technology.

[bb0125] Krippendorff K. (1970). Estimating the reliability, systematic error and random error of interval data. Educ. Psychol. Meas..

[bb0130] Krippendorff K. (2004). Reliability in content analysis: some common misconceptions and recommendations. Hum. Commun. Res..

[bb0135] Kühner, C., Bürger, C., Keller, F., & Hautzinger, M. (2007). [Reliability and validity of the Revised Beck Depression Inventory (BDI-II). Results from German samples]. *Nervenarzt*, *78*(6), 651-656. Doi:10.1007/s00115-006-2098-7 (Reliabilität und Validität des revidierten Beck-Depressionsinventars (BDI-II). Befunde aus deutschsprachigen Stichproben.). 16832698

[bb0140] Lewis J.R. (1991). Psychometric evaluation of an after-scenario questionnaire for computer usability studies: the ASQ. ACM SIGCHI Bull..

[bb0145] Lewis J.R. (1995). IBM computer usability satisfaction questionnaires: psychometric evaluation and instructions for use. Int. J. Hum.-Comput. Interact..

[bb0150] Lewis J.R. (2002). Psychometric evaluation of the PSSUQ using data from five years of usability studies. Int. J. Hum.-Comput. Interact..

[bb0155] Li H., Dong W., Wang Z., Chen N., Wu J., Wang G., Jiang T. (2021). Effect of a virtual reality-based restorative environment on the emotional and cognitive recovery of individuals with mild-to-moderate anxiety and depression. Int. J. Environ. Res. Public Health.

[bb0160] Lindner P., Hamilton W., Miloff A., Carlbring P. (2019). How to treat depression with low-intensity virtual reality interventions: perspectives on translating cognitive behavioral techniques into the virtual reality modality and how to make anti-depressive use of virtual reality-unique experiences. Front. Psychol..

[bb0165] Luborsky L. (1984).

[bb0170] Luborsky L., Popp C., Luborsky E., Mark D. (1994). The core conflictual relationship theme. Psychother. Res..

[bb0175] Mark D.G., Barber J.P., Crits-Christoph P. (2003). Supportive-expressive therapy for chronic depression. J. Clin. Psychol..

[bb0180] Mayring P. (2022).

[bb0185] Mesa-Gresa P., Gil-Gómez H., Lozano-Quilis J.A., Gil-Gómez J.A. (2018). Effectiveness of virtual reality for children and adolescents with autism spectrum disorder: an evidence-based systematic review. Sensors (Basel).

[bb0190] Murtza R., Monroe S., Youmans R.J. (2017).

[bb0195] Nielsen J. (1994). Severity Ratings for Usability Problems. https://www.nngroup.com/articles/how-to-rate-the-severity-of-usability-problems/.

[bb0200] North M.M., North S.M., Coble J.R. (1996). Effectiveness of virtual environment desensitization in the treatment of agoraphobia. Presence: Teleoperators & Virtual Environments.

[bb0205] Osimo S.A., Pizarro R., Spanlang B., Slater M. (2015). Conversations between self and self as Sigmund Freud--A virtual body ownership paradigm for self counselling. Sci. Rep..

[bb0210] Pan X., Hamilton A.F.C. (2018). Why and how to use virtual reality to study human social interaction: the challenges of exploring a new research landscape. Br. J. Psychol..

[bb0215] Paul M., Bullock K., Bailenson J. (2022). Virtual reality behavioral activation for adults with major depressive disorder: feasibility randomized controlled trial. JMIR Mental Health.

[bb0220] Peute L.W., Driest K.F., Marcilly R., Da Costa S.B., Beuscart-Zephir M.-C., Jaspers M.W., Beuscart-Zéphir M.-C., Jaspers M., Kuziemsky C., Nøhr C., Aarts J. (2013). Context Sensitive Health Informatics: Human and Sociotechnical Approaches.

[bb0225] Rutkowski S., Szczegielniak J., Szczepańska-Gieracha J. (2021). Evaluation of the efficacy of immersive virtual reality therapy as a method supporting pulmonary rehabilitation: a randomized controlled trial. J. Clin. Med..

[bb0230] Sansoni M., Malighetti C., Riva G., De In L.T., Paolis P. Arpaia, Sacco M. (2022). Extended Reality XR Salento 2022, Cham.

[bb0235] Sauro J., Lewis J.R. (2016).

[bb0240] Schmidt J., Lamprecht F., Wittmann W.W. (1989). Satisfaction with inpatient management. Development of a questionnaire and initial validity studies. Psychother. Psychosom. Med. Psychol..

[bb0245] Shah L.B., Torres S., Kannusamy P., Chng C.M., He H.G., Klainin-Yobas P. (2015). Efficacy of the virtual reality-based stress management program on stress-related variables in people with mood disorders: the feasibility study. Arch. Psychiatr. Nurs..

[bb0250] Slater M. (2009). Place illusion and plausibility can lead to realistic behaviour in immersive virtual environments. Philos. Trans. R. Soc. Lond. Ser. B Biol. Sci..

[bb0255] Steinert C., Munder T., Rabung S., Hoyer J., Leichsenring F. (2017). Psychodynamic therapy: as efficacious as other empirically supported treatments? A meta-analysis testing equivalence of outcomes. Am. J. Psychiatry.

[bb0260] Sutcliffe A., Gault B. (2004). Heuristic evaluation of virtual reality applications. Interact. Comput..

[bb0265] Szczepańska-Gieracha J., Cieślik B., Serweta A., Klajs K. (2021). Virtual therapeutic garden: a promising method supporting the treatment of depressive symptoms in late-life: a randomized pilot study. J. Clin. Med..

[bb0270] Vanheule S., Desmet M., Rosseel Y., Meganck R. (2006). Core transference themes in depression. J. Affect. Disord..

[bb0275] Wiebe A., Kannen K., Selaskowski B., Mehren A., Thöne A.K., Pramme L., Braun N. (2022). Virtual reality in the diagnostic and therapy for mental disorders: a systematic review. Clin. Psychol. Rev..

[bb0280] World Health Organization (1992).

[bb0285] Yang J.E., Lee T.Y., Kim J.K. (2017). The effect of a VR exercise program on falls and depression in the elderly with mild depression in the local community. J. Phys. Ther. Sci..

