# Supplementary material for: Virtual reality roleplays for patients with depression: A user experience evaluation
Source: Internet Interv. 2024 Jan 19;35:100713. doi: 10.1016/j.invent.2024.100713 (PMC10840098; doi:10.1016/j.invent.2024.100713)
Supplement: Appendix A — Questionnaires during the study. [file mmc1.docx]

**Appendix A**

Questionnaires during the study

| **Questionnaire** | **Measuring point** |  |  |  |
| --- | --- | --- | --- | --- |
|  | Pre | Before Intervention | After Intervention | Post |
| Demographics | x |  |  |  |
| SCL-27 | x |  |  |  |
| BDI-II | x |  |  | x |
| BAI | x |  |  | x |
| MAAS | x |  |  | x |
| PHQ-2 (in VR) |  | x |  |  |
| SAM (in VR) |  | x |  |  |
| ASQ |  |  | x |  |
| UX Interview (semistructured) |  |  | x |  |
| PSSUQ |  |  |  | x |
| ZUF-8 |  |  |  | x |

*Note*: ASQ = After Scenario Questionnaire (Lewis, 1991), BAI = Beck Anxiety Inventory (Beck et al., 1988), BDI-II = Beck Depression Inventory II (Beck et al., 1996), MAAS = Mindful Attention and Awareness Scale (Michalak et al., 2008), PHQ-2 = Patient Health Questionnaire 2 (Löwe et al., 2005), PSSUQ = Post-Study System Usability Questionnaire (Lewis, 1995), SAM = Self-Assessment Manikin (Bradley & Lang, 1994), SCL-27 = Symptom Checklist (Hardt et al., 2004), UX = User Experience, VR = Virtual Reality, ZUF-8 = Satisfaction with Inpatient Care Questionnaire (Schmidt et al., 1989), all questionnaires and the semi-structured UX interview were conducted in German, all questionnaires are self-report measures.

,
