# Supplementary material for: Virtual reality roleplays for patients with depression: A user experience evaluation
Source: Internet Interv. 2024 Jan 19;35:100713. doi: 10.1016/j.invent.2024.100713 (PMC10840098; doi:10.1016/j.invent.2024.100713)
Supplement: Appendix B — Category system. [file mmc2.docx]

**Appendix B**

Category system

| **Category** | **Code** | **Subcategory** | **Definition** |
| --- | --- | --- | --- |
| VR Environment | E1 | Natural engagement | Natural engagement means that interaction in the virtual environment and the virtual environment itself correspond to the user’s expectations about the real world. |
|  | E1a | a. Natural environment and perception | Natural environment and perception means that the virtual environment itself corresponds to the user's expectations about the real world. This includes: environmental consistency, depth perception, physical visibility of (elements of) the virtual environment |
|  | E1b | b. Natural interaction | Natural interactions means that interaction in the virtual environment corresponds to the user's expectations about the real world. This includes: the ability of the representation of the self to interact naturally without restricting normal physical actions; the behavior and task action affordances of objects; the effect of the user’s actions on objects |
|  | E2 | Presence | Presence refers to how much the user feels that they are in the virtual environment, similar to being in a 'real' world. |
|  | E3 | Co-presence | Co-presence refers to the user's subjective feeling that another entity is there. This includes: virtual agents; other users. |
|  | E4 | Simulator sickness | Simulator sickness refers to sensations of physical illness during system use. This includes, among others: nausea, headaches, dizziness, tired or pained eyes, discomfort. |
|  | E5 | Synchronous body movements | Synchronous body movements means that the representation of the self, its field of view, and its behavior in VR stay in synchrony with the user’s head and body movements. |
|  | E6 | Clear entry and exit points | Clear entry and exit points means that the means of entering and exiting from the virtual world are clearly communicated. |
|  | E7 | Navigation and orientation support | Navigation and orientation support means that the user is always able to find where they are in the virtual environment and return to known, preset positions. |
|  | E8 | Clear turn-taking | Clear turn-taking means that it is clearly signaled when it is the system's turn to act and that conventions are established for turn-taking. |
|  | E9 | Consistent departures | Consistent departures means that design compromises are consistent and clearly marked. |
| Device interaction | D1 | User interface design | User interface design refers to how intuitive a system's interface and hardware controls are regarding input and output, interaction, navigation, and design. |
|  | D1a | a. Interface | Interface refers to how intuitive a system's interface is regarding input and output, interaction, navigation, and design. |
|  | D1b | b. Controls | Controls refers to how intuitive a system's hardware controls are regarding input and output, interaction, navigation, and design |
|  | D2 | Learnability | Learnability describes how well the user can learn to use a system while memorability describes how well the user can recall this knowledge later. |
|  | D3 | Switch between real and virtual world | Switch between real and virtual world refers to the extent to which all tasks and information are kept in VR or to which a user needs to take off their VR headset to execute tasks or access information. |
|  | D4 | Headset comfort | Headset comfort refers to headset fit and to how comfortable the headset is to wear. |
|  | D5 | Physical space | Physical space refers to accounting for the real-world physical space the user occupies when interacting with the system. This includes: physical space requirements; physical system components sharing the space (such as stands and cords); real-world objects or people; other real-world circumstances |
|  | D6 | Glitchiness | Glitchiness refers to the occurrence of systematic glitches and other software malfunctions. |
|  | D7 | System setup | System setup refers to the ease of getting the VR system ready for use. This includes: technical setup, device setup, calibration and guardian setup, starting up the desired application. |
| Task-specific | T1 | Fit for purpose | Fit for purpose refers to how fitting the user perceives the system to be with regard to its purpose. This includes: environmental design, interaction design, … |
|  | T2 | Fit for context | Fit for context refers to how fitting the user perceives the system to be with regard to its application in context. This includes both software and hardware components. |
